# Supplementary material for: Refolding Increases the Chaperone-like Activity of αH-Crystallin and Reduces Its Hydrodynamic Diameter to That of α-Crystallin
Source: Int J Mol Sci. 2023 Aug 30;24(17):13473. doi: 10.3390/ijms241713473 (PMC10487585; doi:10.3390/ijms241713473)
Supplement: Supplementary file 1 [file ijms-24-13473-s001.zip › ijms-2532987-supplementary.pdf]

Supplementary materials

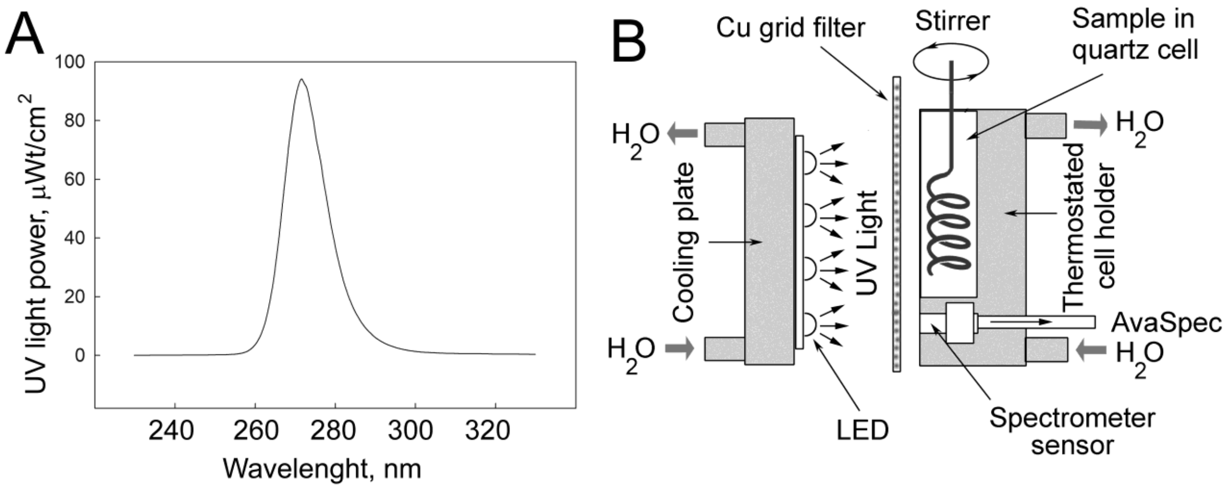

Figure S1. Irradiation of β_L-crystallin solution. A – UV light spectra from LED source. B – Drawing of the irradiance device.

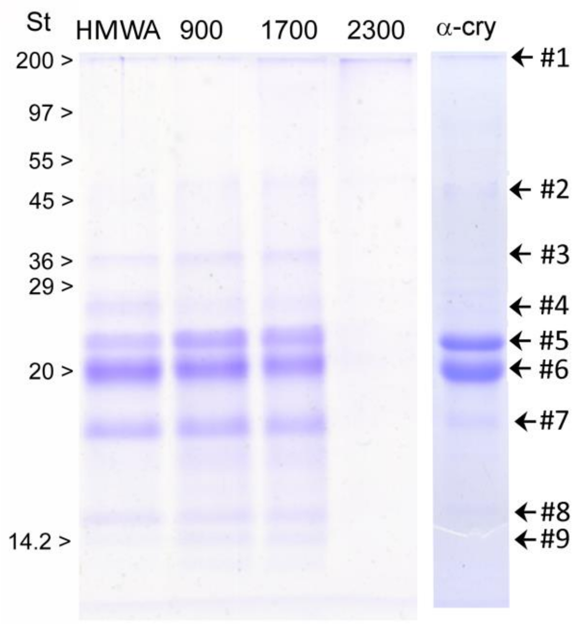

Figure S2. 15% SDS PAGE of HMWA, and HMWAr, α-crystallin. St – protein standards, arrows indicate the polypeptide spots for image analysis.

Table S1. Polypeptide composition of HMWA fractions and a-crystallin

# number	Molecular weight	HMWA	Fraction 900 kDa %	Fraction 1700 kDa %	Fraction 2300 kDa %	α-crystallin %
#1	200	2.5	2.3	2.8	2.8	
#2	38.9	1.2	0.9	1.3	-	
#3	30.5	2.1	3.4	5.1	1.9	
#4	22.4	4.5	1.5	2.7	6.8	
#5	20.1	23.1	25.3	25.6	12.6	30
#6	19.8	48.3	45.6	39.7	52.1	60
#7	19.4	15.0	15.1	16.5	18.6	10
#8	11.5	2.5	3.5	3.5	3.9	
#9	7.6	0.8	2.4	3.0	1.5	

Table S2. Molecular composition of UVβ_L-crystallin formed under set of UV doses.

Control		70 mJ/cm2		140 mJ/cm2		180 mJ/cm2	
MW, kDa	Peak, %	MW, kDa	Peak, %	MW, kDa	Peak,%	MW, kDa	Peak,%
66	99.8	71	99.5	65	98.4	88	97.3
-	-	659	0.3	384	1	1440	1.9
1000	0.2	5500	0.1	781	0.3	2700	0.8

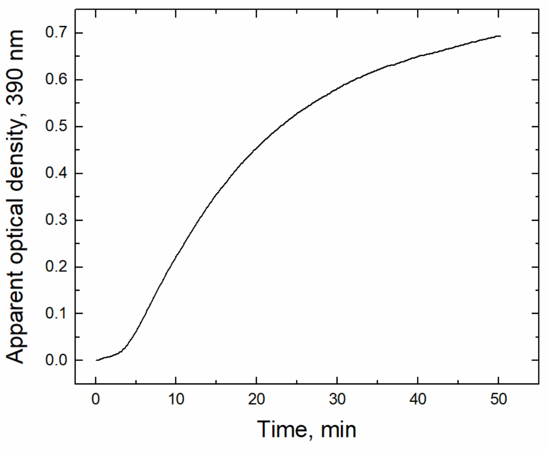

Figure S3. Aggregation of UVβ_L-crystallin at 37 ^{o} C. The summary incident power was 70.0 ± 0.1 μW/cm^2. The irradiation dose was 140 ± 5 mJ/cm^2. Aggregation was monitored with Shimadzu 1240 min spectrophotometer.
